# The effects of high-fat feeding on physical function and skeletal muscle extracellular matrix

**DOI:** 10.1038/nutd.2015.39

**Published:** 2015-12-14

**Authors:** C S Tam, J E Power, T P Markovic, C Yee, M Morsch, S V McLennan, S M Twigg

**Affiliations:** 1 The Charles Perkins Centre and School of Biological Sciences, University of Sydney, Sydney, New South Wales, Australia; 2 Sydney Medical School, and Charles Perkins Centre, University of Sydney, Sydney, New South Wales, Australia; 3 Metabolism and Obesity Services Clinic, Royal Prince Alfred Hospital, Sydney, New South Wales, Australia; 4 Motor Neuron Disease Research Group, Macquarie University, Sydney, New South Wales, Australia; 5 Department of Endocrinology, Royal Prince Alfred Hospital, Sydney, New South Wales, Australia

## Abstract

Skeletal muscle extracellular matrix (ECM) remodelling has been proposed as a feature of the pathogenic milieu associated with obesity and metabolic dysfunction. Whether muscle ECM is associated with impaired physical function in obese conditions is unknown. C57BL/6 mice were fed a high-fat diet (HFD) or chow for 5, 10 and 25 weeks. Non-invasive physiological tests (hang wire, hang mesh and grip strength) to assess neuromuscular function and motor co-ordination were performed. Genes related to ECM structure (COL1, COL3, COL6A2, SPARC), growth factors (TGFB1, TGFB2, CTGF, VEGF) and muscle function (DMD (Dp147), CPN3, DAG1) were measured in gastrocnemius muscle using real-time PCR and COL1, 3 and 6 protein were measured by western immunoblot. Compared with chow, HFD mice had two to six-fold lower muscle strength (hang wire test; raw data and multiplied by body weight) at all time-points (*P*<0.001) and two-fold lower hang mesh and grip strength at 10 weeks (*P*<0.05). At 5 weeks, COL1, COL3 and COL6 gene expression, but not protein levels were three to eight-fold lower in HFD compared with chow. In the HFD group at 5 weeks, greater COL3 and 6 gene expression were associated with poorer hang wire performance. For the first time, our results demonstrate links between muscle ECM structure and physical function in obesity.

## Introduction

Skeletal muscle comprises 40–50% of body weight and is essential for body movement, physical function and insulin-stimulated glucose uptake, all of which are impaired in obesity. An essential component of skeletal muscle is the extracellular matrix (ECM), the non-cellular structure that provides scaffolding for muscle cells. Mounting evidence demonstrates that the skeletal muscle ECM is perturbed under obese and insulin-resistant conditions.^1, 2, 3, 4^ Collagen abundance (total, collagens I and III) is higher in vastus lateralis biopsies from obese insulin-resistant humans^1^ and in diet-induced obese mice, insulin resistance and muscle collagen (III and IV) deposition are rescued by pharmacological or genetic manipulation targeting the ECM.^3,4^ Furthermore, a recently published overfeeding study found dramatic upregulation of mRNA levels in ECM genes (collagens I, III, IV, V and VI and SPARC) in muscle from lean males after moderate weight gain.^2^ This body of evidence suggests a new role for skeletal muscle ECM remodelling in the pathophysiology of obesity and insulin resistance. Whether muscle ECM is associated with impaired physical function in obesity has not yet been investigated.

ECM homeostasis is a balance between synthesis and accumulation of ECM components and ECM breakdown, with clear evidence that ECM regulation strongly affects muscle function.^5^ In normal muscle, the dystrophin–glycoprotein complex provides a stabilizing connection between the actin membrane cytoskeleton and the ECM component laminin. Mutations in genes encoding several dystrophin components result in disruption of this complex leading to muscular dystrophy.^6^ Given the emerging role of muscle ECM remodelling in obesity and insulin resistance, and known structure–function relationships in muscular dystrophies,^7^ we investigated whether muscle ECM gene expression was associated with tests of anaerobic endurance and co-ordination in obese mice.

## Materials and methods

### Mouse models

Six-week-old male C57BL/6 mice were purchased from the Animal Resource Centre (Perth, WA, Australia). To examine the effect of obesity on muscle function and ECM, mice were randomised to chow (12% kcal fat content) or high-fat diet (HFD; 45% kcal fat content) ad libitum for 5, 10 or 25 weeks. The HFD was prepared in-house with a formula based on rodent diet no. D12451; Research Diets (New Brunswick, NJ, USA). Insulin tolerance tests were performed the week prior to termination. After a 4h fast, mice received an i.p. injection of human insulin (Actrapid) at a dose of 0.65IUper kg body weight. Blood glucose levels were measured at 0, 5, 15, 30 and 60min post-insulin administration from 10μl of whole blood using a point of care blood glucose monitoring system (Accu-Check Advantage, Roche Diagnostics, Indianapolis, IN, USA). The excursion in blood glucose over time was calculated as area under the curve (AUC) using the trapezoidal method. For each curve generated, the change in AUC was compared with the AUC obtained for the chow group (normalised to 100%). At termination, gastrocnemius muscle was excised, weighed and snap frozen in liquid nitrogen for gene expression and protein analysis. Six independent experiments were performed using this protocol and the data were combined across experiments. At 5, 10 and 25 weeks sample sizes were 9 chow vs 12 HFD, 11 chow vs 13 HFD and 11 chow vs 19 HFD. Investigators were not blinded to the intervention. All mice in the HFD group met the inclusion criteria of gaining ⩾5g the average weight gained by the chow group. This study was approved by the Animal Ethics Committee of Sydney South West Area Health Services, Australia.

### Non-invasive muscle function tests

To assess muscle function, a series of non-invasive motor tests (hang wire, hang mesh, grip strength) were performed in the week prior to termination using standard operating protocols from the Treat-NMD Neuromuscular Network.^8^ The hang wire tests whole body anaerobic muscular endurance and co-ordination. Outputs include the number of falls and reaches (up to a maximum of 10) and the duration of hang time (up to a maximum of 180s). An aggregate score from the number of falls, reaches and time metrics was derived using the formula: ([10 falls+reaches+1] × time). The hang mesh was performed to test four limb anaerobic muscular endurance. Grip strength was performed to test maximal isometric forelimb grip strength. To adjust for potential effects of body weight on test performance, results from hang wire and hang mesh tests were multiplied by body weight. Muscle function tests are further described in Supplementary Data.

### Real-time PCR measures

Total RNA was extracted using TRI reagent (Sigma-Aldrich, St Louis, MO, USA). RNA amount and quality were determined using a Nanodrop spectrophotometer (Nanodrop Technologies, Wilmington, DE, USA). Total RNA (1μg) was then reverse-transcribed to complementary DNA using 10pmol of oligo(dT)_12–18_ primer (Invitrogen, Carlsbad, CA, USA) and SuperScript III reverse transcriptase (Invitrogen). Expression of genes related to the ECM (collagen (*COL)1*, *COL3*, *COL6A2,* secreted protein acidic and rich in cysteine (*SPARC*)), growth factors ((transforming growth factor (*TGFB)1*, *TGFB2*, connective tissue growth factor (*CTGF*, also known as CCN2), vascular endothelial growth factor (*VEGF*)) and muscle function (dystrophin (*DMD* (Dp147)), calpain 3 *(CAPN3)*, β-dystroglycan (*DAG1)*) were measured using SensiMixII (Bioline, Alexandria, NSW, Australia) and SYBR green fluorophore (Invitrogen). Primer sequences are listed in Supplementary Table 1. Results are expressed as relative change from chow-fed mice calculated using the delta–delta method and corrected for the housekeeper 18S (23).

### Protein analysis

For measurement of collagens 1, 3 and 6, 25μg of total protein per sample was loaded on 4–15% Mini-Protean TGX gels (BioRad/BioRad Laboratories, Hercules, CA, USA). Membranes were blocked for 1.5h at room temperature with 5% skim milk in TBST, and then incubated overnight at 4°C with antibodies against COL1 (AbCam, Cambridge, MA, USA; AB765P, 1:500), COL3 (AbCam, AB7778, 1:500) or COL6 (AbCam, AB6588, 1:5000). Goat anti-rabbit secondary antibody (Sigma, Castle Hill, NSW, Australia; AD545, 1:7000) was applied for 2h at room temperature. Bands were imaged with ChemiDoc MP system and quantified using ImageLabTM software (Version 5.2, BioRad). Bands were normalized to total lane protein (Ponceau staining) and inter-gel variation was corrected using an internal control run across all gels.

### Statistical analyses

Statistical analyses were performed using SPSS Version 22 (IBM Corporation, Armonk, NY, USA) and GraphPad Prism 6 (GraphPad Software Inc., La Jolla, CA, USA) and data presented as mean±s.d. At each time-point, Mann–Whitney *U*-tests were performed to investigate differences in physical function and gene expression between chow and HFD groups and correlations were performed using Pearson's coefficient.

## Results

### Reduced physical function in high-fat fed mice

**Figure 1:**
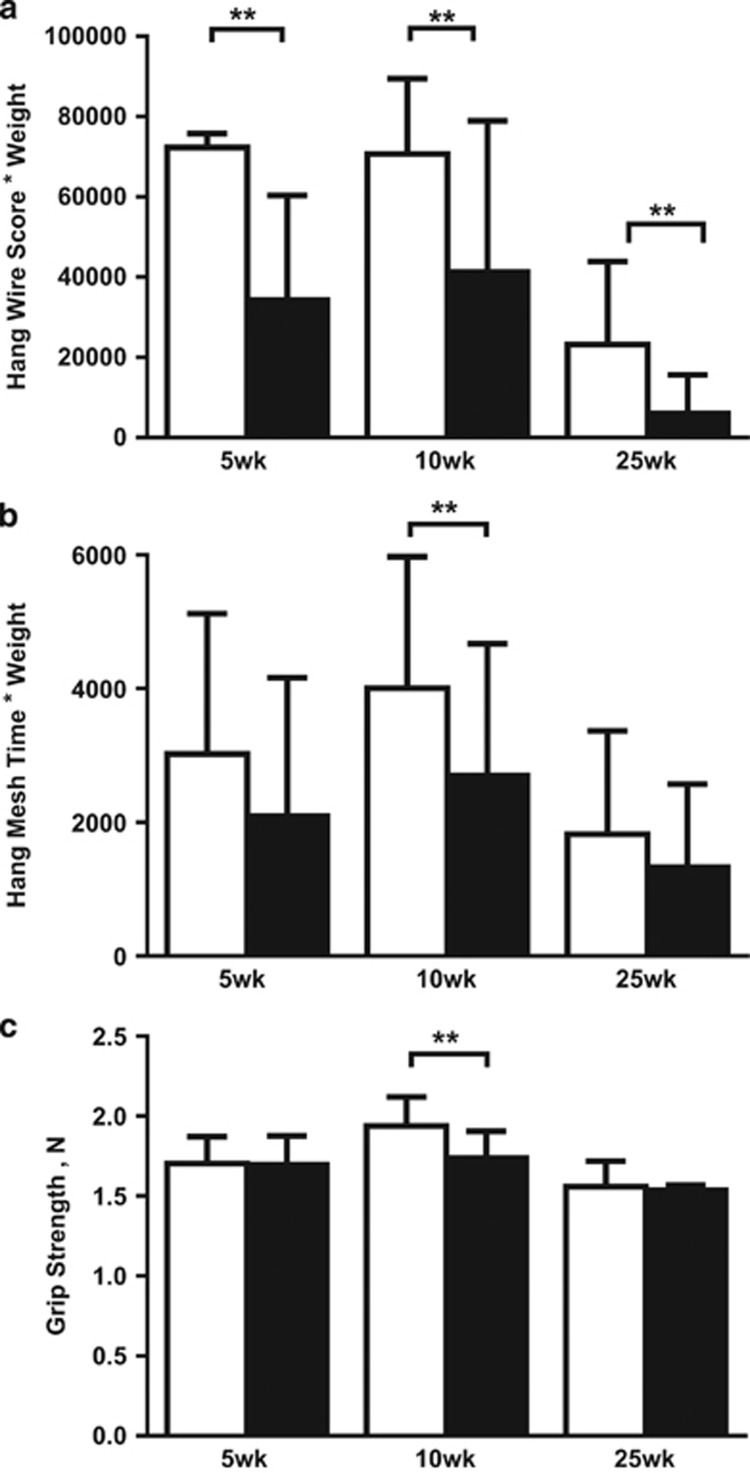
Changes in muscle function after 5, 10 and 25 weeks of high-fat diet. Muscle function tests include the (**a**) hang wire, (**b**) hang mesh and (**c**) grip strength test. To account for potential effects of body weight, hang wire and hang mesh results are multiplied by body weight. At each time-point, Mann–Whitney *U*-tests were performed to examine differences between chow and high-fat fed mice. Data are presented as mean±s.e.m. **P<0.001; Unfilled bars, chow; filled bars, high-fat diet.

As expected, HFD mice were significantly heavier than chow-fed mice at all time-points. Fasting blood glucose levels and excised gastrocnemius weight were not different between groups (Supplementary Table 2). At all time-points, HFD mice had significantly greater numbers of falls of the wire, less numbers of reaches and less hanging time on the wire compared with chow-fed mice (all *P*<0.001; data not shown). HFD mice had two to six-fold lower hang wire scores (raw data and values adjusted for body weight) compared with chow-fed mice (all *P*<0.001; Figure 1). At 10 weeks, HFD mice also had lower hang mesh score (median: chow=4733; HFD= 2207; *P*=0.04) and grip strength (median: chow=2.0N; HFD=1.7N; *P*=0.0003) compared with chow-fed mice. There were no significant correlations between hang wire, hang mesh and grip strength scores measured in the same mice at all time-points (data not shown).

### ECM gene expression and protein levels

**Figure 2:**
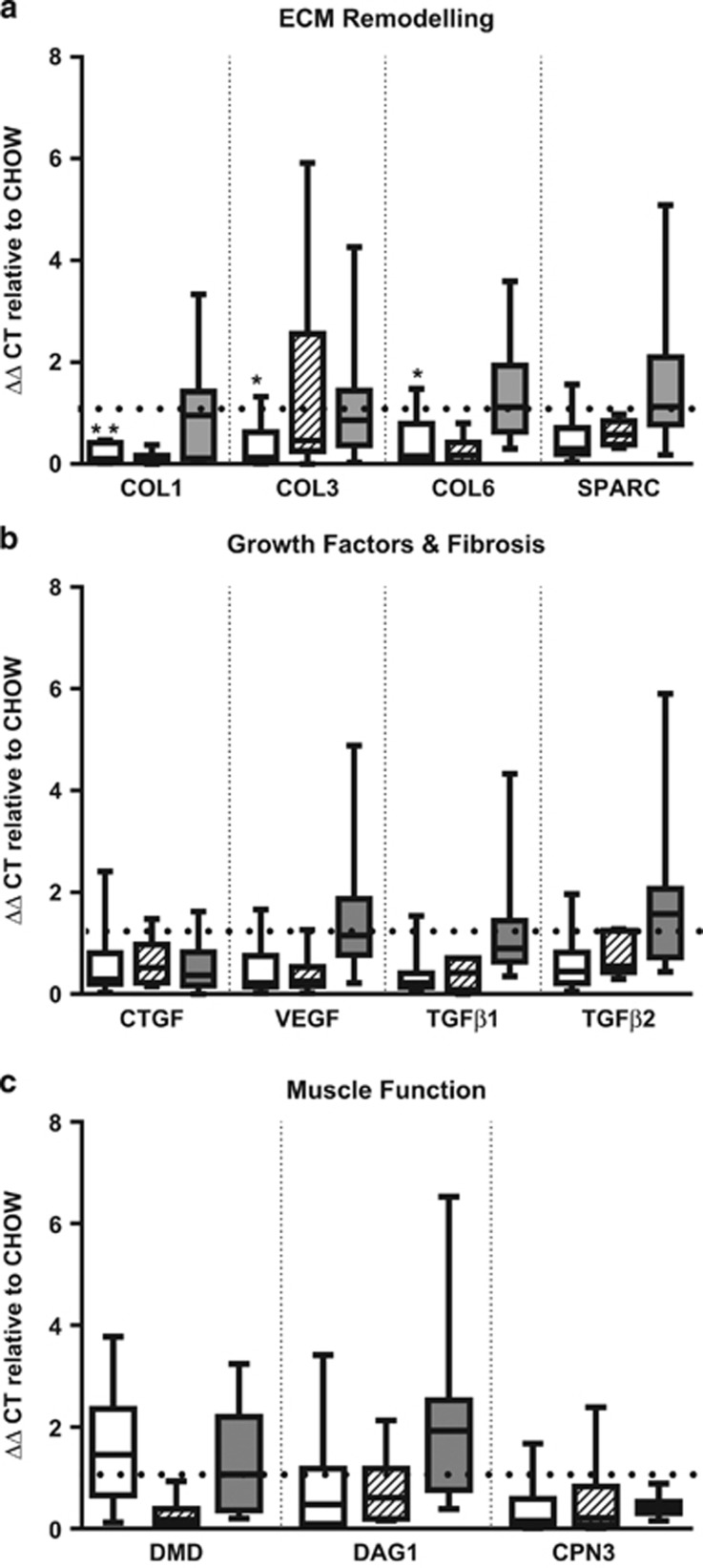
Changes in gastrocnemius mRNA levels in genes related to (**a**) extracellular matrix remodelling, (**b**) extracellular matrix gene regulation and (**c**) muscle function. Gene expression was calculated using ΔΔCT and expressed as CHOW=1 (represented by dotted line). At each time-point, Mann–Whitney *U*-tests were performed to examine differences between chow and high-fat fed mice. Empty bars, 5 weeks; Striped bars, 10 weeks; Grey bars, 25 week time-point. CAPN3, calpain 3; COL, collagen; CTGF, connective tissue growth factor; DAG1, β-dystroglycan; DMD, dystrophin; SPARC, secreted protein and rich in cysteine; TGF, transforming growth factor; VEGF, vascular endothelial growth factor. *P<0.05, **P<0.001.

COL1 (*P*=0.002), COL3 (*P*=0.02) and COL6 (*P*=0.03) mRNA levels were eight, three and four-fold lower, respectively, after 5 weeks of HFD, compared with chow (Figure 2). These differences in COL1, 3 and 6 were not observed by protein analyses (Supplementary Figure 1). No other significant differences were observed for the rest of the genes measured.

### Associations between physical function and muscle ECM

Next we examined associations between measures of physical function (hang wire, hang mesh, grip strength) and ECM gene expression and protein levels. Interestingly, only in the HFD group, higher COL3 (*r*=−0.69, *P*=0.02) and COL6 gene expression (*r*=−0.71, *P*=0.03) were associated with poorer hang wire performance at 5 weeks (Supplementary Figure 2). These associations were not seen in chow-fed mice or after 10 and 25 weeks HFD. No associations were found between hang wire or grip strength with ECM steady state mRNA levels, and no associations were observed between physical function tests and COL1, 3 and 6 protein levels at 5 weeks.

## Discussion

This study examined potential links between muscle ECM gene expression and impaired physical function in obese mice. We found that mice fed HFD for 5, 10 and 25 weeks had impaired anaerobic endurance and co-ordination as measured by hang wire test. These findings are in keeping with results in humans from large cohort studies demonstrating the strong relationship between elevated BMI and functional decline, where being overweight or obese is associated with impairments in activities of daily living.^9, 10, 11^ At 10 weeks, we also found lower hang mesh and grip strength performance in the HFD group, which was not evident at 25 weeks, suggesting a potential compensation effect with a longer duration high-fat feeding.

Intriguingly, muscle ECM remodelling genes (COL1, 3 and 6) were lower after 5 weeks HFD with no changes at subsequent time-points. Our findings are somewhat in contrast to previous studies demonstrating greater collagen deposition in skeletal muscle from obese insulin-resistant adults^1^ and increased muscle collagens 1 and 3 expression after 20 weeks HFD in mice.^3^ The mice used in these studies did not exhibit significant impairments in insulin sensitivity or glucose homeostasis and if the HFD was successful in generating insulin resistance as in previous mouse studies,^3,4^ the ECM markers measured may have better mimicked the human condition. However, interestingly we found associations between muscle ECM structure and function, with reduced physical function (hang wire test) associated with greater COL3 and 6 expression only in the HFD group at 5 weeks; this finding was not confirmed at the protein level. The significance of the association seen at the level of gene expression only is unclear. It may be that high-fat feeding affects muscle structure gene regulation and muscle function, each in parallel, as we have observed in this study, rather than structural changes mediating functional changes. Future experiments using alternative mouse strains, greater percent fat diets (>45%) and shorter time courses of high-fat feeding (<5 weeks) may assist in further elucidating possible functional consequences of perturbed muscle ECM. Alternatively, our finding of reduced COL1, COL3 and COL6 muscle expression after short-term HFD may indicate an imbalance in ECM homeostasis. Investigations examining factors that promote or inhibit the synthesis or degradation of the ECM such as matrix metalloproteinases may provide further insight. In conclusion, impaired physical motor skills in high-fat fed mice were found in this study. The impairment was associated with greater COL3 and COL6 gene expression in the gastrocnemius muscle only after short-term HFD, suggesting that muscle ECM expression can be linked to impaired physical function. The muscle ECM may be a potential target for enhancing physical function under obese conditions.

## Supplementary Material

Supplementary Informations
