# Supplementary material for: The effects of high-fat feeding on physical function and skeletal muscle extracellular matrix
Source: Nutr Diabetes. 2015 Dec 14;5(12):e187–. doi: 10.1038/nutd.2015.39 (PMC4735053; doi:10.1038/nutd.2015.39)
Supplement: Supplementary Informations [file nutd201539x1.docx]

**Supplementary Data**

**Methods for muscle function tests**

The *hang wire* tests whole body anaerobic muscular endurance and coordination ([22](#_ENREF_22)). Mice were positioned such that they hung by their forelimbs from a 55cm long, 2mm thick wire suspended 35cm above a cushioned surface. Mice could then remain hanging, move to either end of the wire, or fall from the wire. The number of times that the end of the wire was reached and the number of times fallen from the wire were recorded, with a maximum of 10 falls or 180 seconds reached, before the test was stopped. An aggregate score from the falls, reaches and time metrics was derived using the formula: ([10-falls+reaches+1]*time).

The *hang mesh* was performed to test four limb anaerobic muscular endurance ([21](#_ENREF_21)). Mice were placed inside a transparent 12 cm diameter tube of which one end was sealed with 1 cm gauge mesh, designed so that the mesh was the only surface available inside the tube. The tube was then agitated such that the mouse instinctively gripped the mesh, and immediately inverted at 35cm above a cushioned surface. The timer was activated upon inversion and the best of 3 attempts was recorded (time in s) with a maximum time of 180s.

*Grip strength* was performed to test maximal isometric forelimb grip strength ([20](#_ENREF_20)): mice were allowed to form a pronated forelimb grip on the horizontal wire attachment of a horizontally oriented force transducer (Chatillon, DFIS-2). Mice were pulled horizontally away from the transducer with a constant motion and peak force coincided with the release of grip. Force (newtons) was recorded for 3 attempts.

**Supplementary Table 1. Primer Sequences**

| **Symbol** | **Gene** | **Sequence** |
| --- | --- | --- |
| *COL1* | Collagen 1 | (Forward) ccccgggactcctggactt  (Reverse) gctccgacacgccctctctc |
| *COL3* | Collagen 3 | (Forward) cctggagcccctggactaatag  (Reverse) gcccattgcaccaggttct |
| *COL6a2* | Collagen 6 | (Forward) gaacttccctgccaaacaga  (Reverse) caccttgtggaagttctgctc |
| *SPARC* | Secreted protein and rich in cysteine | (Forward) GTCCACAAGCACGAGGAGAT  (Reverse) CTCCAAACCACCAGGGGAAA |
| *TGFb1* | Transforming growth factor *b* 1 | (Forward) tggagcaacatgtggaactc  (Reverse) gtcagcagccggttacca |
| *TGFb2* | Transforming growth factor *b* 2 | (Forward) gctaatgttgttgccctcct  (Reverse) gcagcaattatcctgcacatt |
| *CTGF* | Connective tissue growth factor | (Forward) gaagggcaaaaagtgcatcc  (Reverse) cagttgtaatggcaggcac |
| *VEGF* | Vascular endothelial growth factor | (Forward) TTACTGCTGTACCTCCACC  (Reverse) ACAGGACGGCTTGAAGATG |
| *DMD*  *(Dp147 isoform)* | Dystrophin | (Forward) TCTCATCGTACCTAAGCCTC  (Reverse) CAGTGCCTTGTTGACATTGTTCAG |
| *DAG1* | β-dystroglycan | (Forward) TAGAGAAGACCAAGGGACAGT  (Reverse) GTGCATGGGGTAGAGGTCAG |
| *CAPN3* | Calpain 3 | (Forward) GTGGACACCCAAGTGGCATC  (Reverse) TGCGGAGCTGCTCAAATGTC |
| *18S* | 18S | (Forward) cggctaccacataccaaggaa  (Reverse) gctggaattaaccgcggct |

**Supplementary Table 2. Effects of high-fat diet on physiological variables**

|  | Time-point, week | Chow | HFD |
| --- | --- | --- | --- |
| Δ Body weight, g | 5 | 4.5 ± 1.4 | 12.1 ± 3.0** |
|  | 10 | 5.9 ± 1.9 | 16.5 ± 3.5** |
|  | 25 | 11.7 ±4.4 | 28.1 ± 4.7** |
|  |  |  |  |
| Fasting blood glucose, mmol/L | 5 | 7.5 ± 1.5 | 7.5 ± 1.4 |
|  | 10 | 7.3 ± 1.5 | 7.1 ± 1.0 |
|  | 25 | 9.4 ± 3.2 | 10.0 ± 1.8 |
|  |  |  |  |
| Insulin resistance by tolerance test, glucose AUC, %change from CHOW^a^ | 5 | 100 ± 66 | 96 ± 42 |
|  | 10 | 100 ± 29 | 100 ± 50 |
|  | 25 | 100 ± 70 | 102 ± 63 |
|  |  |  |  |
| Excised gastrocnemius weight, mg | 5 | 156 ± 5 | 169 ± 4 |
|  | 10 | 138 ± 3 | 123 ± 1 |
|  | 25 | 168 ± 2 | 160 ± 1 |

*P<0.05; **P<0.001. P values represent differences between Chow and HFD groups (Independent Sample T-tests). Data presented as mean ± SD.

^a^ Insulin tolerance tests were performed using intraperitoneal injection of human insulin (Actrapid; 0.65 IU/kg body weight). Blood glucose levels were measured at 0, 5, 15, 30 and 60 minutes post-insulin administration and the excursion in blood glucose over time calculated as AUC using the trapezoidal method. For each curve generated, the change in AUC was compared with the AUC obtained for the chow group (normalized to 100%).

**Supplementary Figure 1. Collagen 1, 3 and 6 levels in chow (n=8) and high-fat fed mice (n=9) at 5 weeks. a)** Representative blots for COL1, 3 and 6 from chow and HFD groups and **b)** Quantification of band intensity. Empty bars represent chow and filled bars represent high-fat fed animals. Band intensity was normalized to total protein (Ponceau staining). Data are presented as mean±SEM.


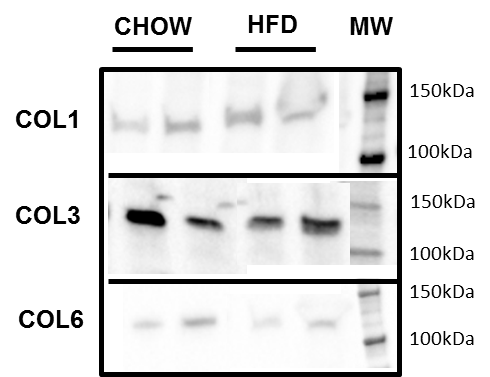


**a)**

**b)**

**Supplementary Figure 2. Associations between a) COL3 and b) COL6mRNA levels and hang wire score (multiplied by body weight) in high-fat fed mice at 5 weeks.**
